# Using Open-Access Data to Explore Relations between Urban Landscapes and Diarrhoeal Diseases in Côte d’Ivoire

**DOI:** 10.3390/ijerph19137677

**Published:** 2022-06-23

**Authors:** Vitor Pessoa Colombo, Jérôme Chenal, Brama Koné, Martí Bosch, Jürg Utzinger

**Affiliations:** 1 School of Architecture, Civil and Environmental Engineering, École Polytechnique Fédérale de Lausanne, 1015 Lausanne, Switzerland; jerome.chenal@epfl.ch (J.C.); marti.bosch@epfl.ch (M.B.); 2 Centre Suisse de Recherches Scientifiques en Côte d’Ivoire, Abidjan 01 BP 1303, Côte d’Ivoire; brama.kone@csrs.ci; 3 Swiss Tropical and Public Health Institute, 4123 Allschwil, Switzerland; juerg.utzinger@swisstph.ch; 4 University of Basel, 4001 Basel, Switzerland

**Keywords:** African cities, Cote d’Ivoire, diarrhoea, landscape ecology, open-source software, urban planning

## Abstract

Unlike water and sanitation infrastructures or socio-economic indicators, landscape features are seldomly considered as predictors of diarrhoea. In contexts of rapid urbanisation and changes in the physical environment, urban planners and public health managers could benefit from a deeper understanding of the relationship between landscape patterns and health outcomes. We conducted an ecological analysis based on a large ensemble of open-access data to identify specific landscape features associated with diarrhoea. Designed as a proof-of-concept study, our research focused on Côte d’Ivoire. This analysis aimed to (i) build a framework strictly based on open-access data and open-source software to investigate diarrhoea risk factors originating from the physical environment and (ii) understand whether different types and forms of urban settlements are associated with different prevalence rates of diarrhoea. We advanced landscape patterns as variables of exposure and tested their association with the prevalence of diarrhoea among children under the age of five years through multiple regression models. A specific urban landscape pattern was significantly associated with diarrhoea. We conclude that, while the improvement of water, sanitation, and hygiene infrastructures is crucial to prevent diarrhoeal diseases, the health benefits of such improvements may be hampered if the overall physical environment remains precarious.

## 1. Introduction

Diarrhoeal diseases still pose a considerable public health problem. Although diarrhoea is preventable and treatable, it remains one of the main causes of death worldwide, especially among young children and the elderly in sub-Saharan Africa [1]. With over 800,000 deaths attributed to inadequate water, sanitation and hygiene (WASH) in 2016, diarrhoea is the main component of the disease burden from unsafe WASH [2].

It is widely acknowledged that safe access to well-managed WASH services is key to interrupting the transmission pathways of pathogens causing diarrhoeal diseases [3,4,5]. Nonetheless, important research gaps remain, notably regarding potential risk factors related to the physical environment. For example, although previous studies have explained the relevance of understanding spatial patterns of diarrhoea [6,7], little is known about the impact of landscape patterns—i.e., the form and composition of the physical environment—on the occurrence of diarrhoeal diseases. Besides socio-economic and biological factors, the physical environment can also be considered as a key health determinant [8,9]. Moreover, understanding the relationship between diarrhoea and the spatial characteristics of human settlements becomes even more relevant in the case of child health, considering that the main exposure pathway for toddlers is hand-to-mouth contamination [10], which relates directly to the living environment.

In this study, we adopted a landscape ecology perspective [11] to deepen the current knowledge on risk factors for diarrhoea, by advancing “urban landscapes” as the key exposures. We purposively refer to urban landscapes in the plural, positing that they result from specific, local socio-spatial arrangements that come to bear in different forms. Indeed, cities are “patchy” ecosystems [12], formed by different types of land use and occupation that materialize into extremely diverse urban habitats—arguably, with some of these habitats being more healthy than others. In this sense, landscape ecology methods are instrumental to analyse the form and composition of urban habitats, by addressing landscape as spatial mosaic of discrete land use/land cover “patches” [11,13,14].

Thanks to advances in geospatial technologies and access to georeferenced health data, spatial patterns of disease have been extensively studied [7,15], while mathematical models have been developed to analyse and predict the spatiotemporal spread of infectious diseases [16]. However, most studies conducted at large geographic scales (e.g., national, or metropolitan scales) focus only on disease clustering, and inadequately address space as an abstract, homogenous entity. In fact, variables related to the physical environment (e.g., entropy levels of the built environment, density or land-use) are rarely integrated in large-scale studies—exceptions occur when both health and settlement data are available at a high spatial resolution, or are collected directly by the investigators [17,18].

The lack of high-resolution data systematically collected over large areas constitutes a barrier to ecological studies that aim to explore risk factors derived from the physical environment [19], especially in low- and middle-income countries [20]. Such research barriers are being overcome with rapidly growing open-access data portals (e.g., OpenStreetMap and Humanitarian Data Exchange) and the increased contribution of open science projects that freely share their data. There is a high potential to combine these openly accessible datasets to deepen the understanding of how the physical environment relates to health. Yet, there is a paucity of studies that use open-access data to investigate this relationship at a large geographic scale. Such analyses could provide a useful framework, given their low cost and high reproducibility.

Moreover, understanding how the physical environment affects health is key for decision-making, notably in contexts marked by high levels of socio-spatial segregation that lead to disparate health outcomes [9]. Indeed, in cities characterised by inequitable spatial development, the spatial dimension of disease must be addressed to properly identify priorities and to tailor targeted interventions. In sub-Saharan Africa, given the dynamics and intensity of recent urbanisation trends [21], decision makers would benefit from a deeper understanding of the relations between urban landscape patterns and diarrhoeal diseases.

The purpose of this study was to explore the possible relations between urban landscapes and diarrhoea, addressing the hypothesis that specific, spatial patterns of urban settlements can be associated with the prevalence of diarrhoea. In this way, the spatial characteristics of the physical environment were defined as key variables of exposure. At the same time, our study put forward an analytical framework that was strictly based on open-access data and open-source software. We focused on Côte d’Ivoire, a West African country that faces considerable challenges related to rapid urbanisation and has a high burden of diarrhoeal diseases, which are among the country’s top 10 causes of death [22]. In addition, Ivorian cities are marked by contrasting landscapes, as economic growth has failed to keep pace with the rapid urban and demographic growths [23], while transposed town planning models have failed to address local socio-economic needs [24]. Notably, access to water and sanitation services remain a great challenge. Between 2000 and 2020, access to basic water services in Ivorian cities decreased from 91 to 85%, while 52% of the urban population still lacked access to basic sanitation in 2020 [25]. The concentration of demographic growth around a few cities (“urban primacy”) has certainly exacerbated the issues related to the lack of basic services, as these cities have become saturated and witnessed the emergence of precarious, informal settlements [23]. In 2014, over half of the urban population in Côte d’Ivoire lived in slums [26], i.e., deprived settlements where the physical environment itself exacerbates the risk of several illnesses, including diarrhoeal diseases [9,27].

## 2. Materials and Methods

### 2.1. Datasets

This cross-sectional, ecological study combined open-access data on health, land cover, and basic infrastructures, readily obtained from different sources (Table 1). Geospatial data on the occurrence of diarrhoea among children under the age of five years can be obtained from the Demographic and Health Surveys (DHS) programme in vector format [28]. The DHS provides anonymised survey data at the individual and household levels that include cases of diarrhoea that had occurred in the 2 weeks preceding their survey. The DHS also provides data on access to WASH facilities and education, which were relevant to the current analysis. DHS data can be georeferenced by linking the observations to the point locations of survey clusters. To ensure anonymity, these locations do not correspond to the precise locations of participating households, but rather to the mean location of households belonging to a same cluster—i.e., for every cluster of surveyed households, there is one single GPS position that is attributed to all households belonging to the same cluster. For the datasets used in this analysis [29,30,31,32], there are 351 clusters distributed across Côte d’Ivoire, for a total of 9686 surveyed households. These clusters contain a median number of 27 households, or 134 people. Models of weather conditions can be obtained from Terraclimate in raster format, at a spatial resolution of ~4 × 4 km [33], and these data were included in our study because climatic conditions can be associated with diarrhoea [34]. Night illumination was used as a proxy for the presence of urban infrastructures, and can be obtained in raster format (500 × 500 m) from the National Aeronautics and Space Administration’s (NASA) Earth Observatory [35]. Land cover data can be obtained in raster format (300 × 300 m) from the European Space Agency’s Land Cover Climate Change Initiative Project (ESA Land Cover CCI) [36]. Population estimates at high spatial resolutions are provided by WorldPop [37], also in raster format (100 × 100 m). Finally, vector data on mobility infrastructures can be obtained from OpenStreetMap.

### 2.2. Data Pre-Processing

Combining health and environmental data is often a challenge, considering the differences in terms of the spatial and temporal resolutions of openly available data [38]. In this study, the areal units used to aggregate data were based on the geolocations of DHS clusters, which had the coarsest spatial resolution. All data processing steps were done in Python language, using the Jupyter computing environment. The following packages were used to process and visualise the data: PyLandStats 2.3.0 [39], rasterstats 0.15.0 [40], rasterio 1.2.9 [41], earthpy 0.9.2 [42], Fiona 1.8.20 [43], pandas 1.3.4 [44], geopandas 0.9.0 [45], numpy 1.20.3 [46], statsmodels 0.13.0 [47], pysal 2.5.0 [48], matplotlib 3.4.3 [49], and seaborn 0.11.2 [50].

**Figure 1:**
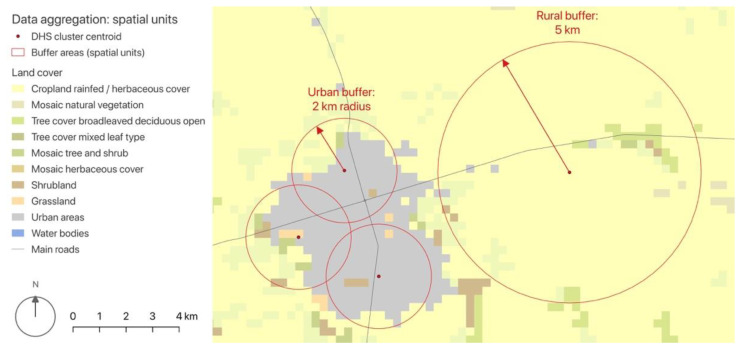
Buffer areas (red circles generated from DHS clusters’ centroids) were used as reference areas to calculate landscape metrics. Elaborated by the authors with QGIS, from: DHS [28] and ESA Land Cover CCI [36]. © ESA Climate Change Initiative—Land Cover led by UCLouvain (2017).

The different data layers were harmonised spatially and temporally based on the buffer areas generated from DHS clusters, hereinafter called “spatial units”. Based on previous studies [51], we generated circular buffer zones originating from each cluster centroid, with radii based on the geographic blur determined by the DHS data anonymisation protocol: urban clusters had a buffer radius of 2 km, while rural clusters had a buffer radius of 5 km (Figure 1). The different data layers were aggregated at the cluster level: environmental data were aggregated based on the respective buffer areas, while DHS survey data were aggregated based on the clusters’ unique identifiers. In this way, for each spatial unit we obtained the following: (i) the prevalence of diarrhoea among children under the age of five; (ii) the proportion of the cluster’s population with access to at least “basic” water and sanitation, as defined by the World Health Organisation (WHO) and the United Nations Children’s Fund (UNICEF) Joint Monitoring Programme [25]; (iii) the proportion of the cluster’s female population who never attended school; (iv) local climatic conditions; (v) a list of landscape metrics derived from remotely sensed data (NASA and ESA Land Cover CCI), hybrid models (WorldPop), and volunteered geographic information (OpenStreetMap). Table 2 shows the variables obtained from this pre-processing in more detail.

Landscape metrics have been widely used to quantify and describe spatial patterns of “patches” of similar land cover categories [11,14,39], and thus are useful to analyse spatial patterns of urban settlements. These metrics were key features to our study, allowing us to relate the prevalence of diarrhoea (dependent variable) to indicators describing the form and composition of urban settlements (independent variables). We used the Python package *PyLandStats* [39] to calculate a series of landscape metrics for each land cover class contained within each spatial unit’s perimeter (Table 2), based on the data provided by ESA’s Land Cover Climate Change Initiative Project [36]. The metrics employed here—i.e., the proportion, edge and shape index of land cover patches—were based on previous studies that referred to land cover data to analyse spatial patterns of urban settlements [14] and environmental determinants of disease [18].

**Figure 2:**
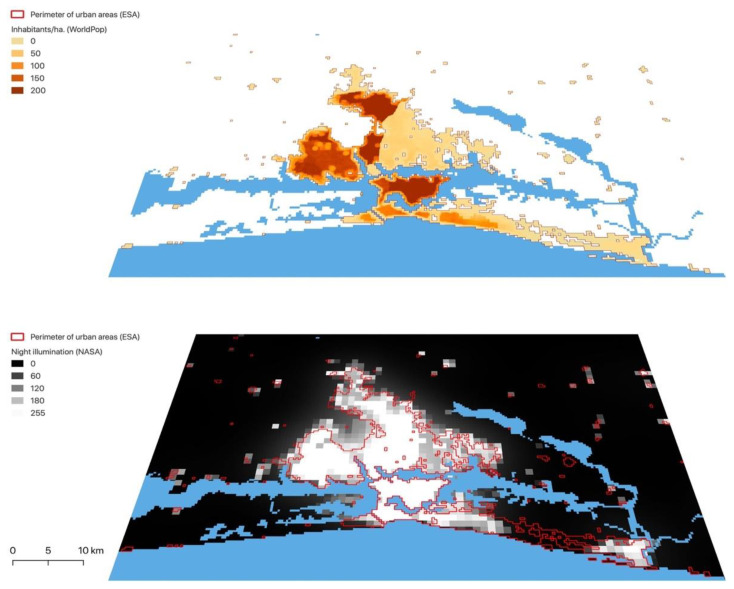
Layers used to reclassify urban patches based on demographic density and night illumination (zoom on Abidjan’s metropolitan area). Elaborated by the authors with QGIS, from: NASA [35], ESA Land Cover CCI [36] and WorldPop [37]. © ESA Climate Change Initiative—Land Cover led by UCLouvain (2017).

**Figure 3:**
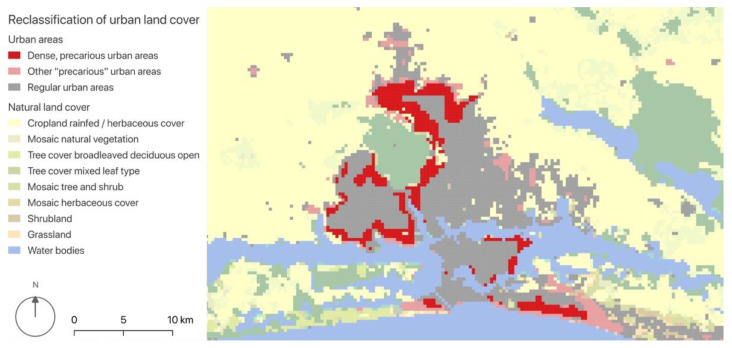
Reclassified urban patches (zoom on Abidjan’s metropolitan area). Elaborated by the authors with QGIS, from: NASA [35], ESA Land Cover CCI [36] and WorldPop [37]. © ESA Climate Change Initiative—Land Cover led by UCLouvain (2017).

**Figure A1:**
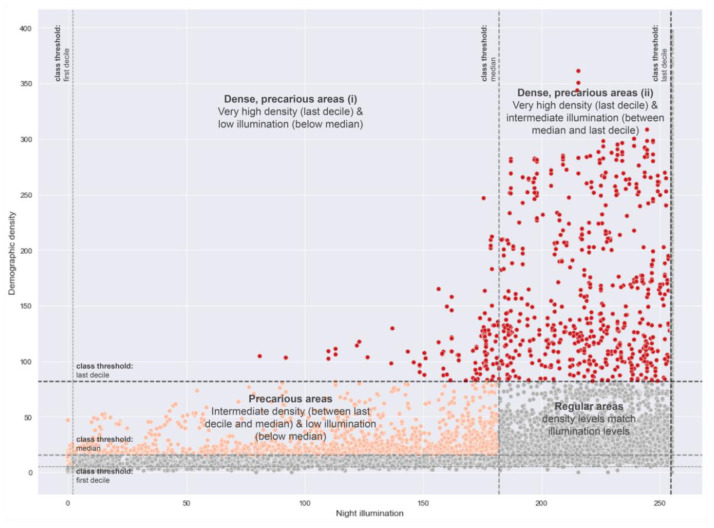
Scatterplot showing the reclassification of urban pixels (300 × 300 m) according to demographic density and night illumination. Elaborated by the authors.

We added a level of detail to our landscape metrics by reclassifying patches originally categorised as “urban”, based on the levels of night illumination and demographic density (Figure 2 and Figure 3). Our study used the intensity of night illumination as a proxy for the presence of urban infrastructures—or, in other words, for the “quality” of urbanisation. Moreover, the quality of illumination affects the use of WASH amenities, especially by females [52], and thus may impact the risk of diarrhoea. We defined “precarious” and “regular” urban areas, building on the hypothesis that, if basic infrastructures are present, the level of illumination follows the level of demographic density. In this sense, urban pixels were considered “precarious” if they presented a high demographic density but a low (or relatively low) intensity of night illumination; on the other hand, “regular” urban patches had night illumination levels that matched the demographic density. This categorisation was done based on quantiles (Figure A1, Appendix A): for each urban pixel, if its density value was situated in a higher quantile than its night illumination value, it was considered “precarious”.

Another indicator of the quality of urbanisation was the presence of roads, for which we calculated two indicators: road availability (km of roads per ha of built-up area) and linearity (ratio between edge length and linear distance between the two vertices of the same edge). The latter also served as an indicator of the urban form.

Finally, based on the literature, a selection of features that have been associated with diarrhoea were included as control variables. Basic water and sanitation services are key to prevent diarrhoea [2], and were therefore included. Access to these services was measured using the DHS household datasets [30]. Maternal education has been associated with a lower risk of diarrhoea [53]; at the same time, it has been related with reporting bias, as households with higher levels of maternal education have shown increased rates of reported child diarrhoea [54]. A proxy variable of maternal education (i.e., women’s educational attainment) was therefore added, based on individual DHS data [31]. Climatic conditions also have been associated with diarrhoea [34], but here they showed no significant correlation (see Table A3, Appendix D). Hence, these data were discarded (for more details, see the computer code section at the end of this article).

To preserve some level of detail in the data, the variables resulting from the aggregation at the cluster level consisted of proportions (percentage) rather than simple means or medians. For example, demographic density in each spatial unit was given by the ratio between the number of built-up pixels classified as “dense” (with a value higher than the statistical series’ median) and the total number of built-up pixels contained in the respective spatial unit.

By the end of the pre-processing, we obtained a geo-dataset with 351 spatial units. Each spatial unit had three control variables (water, sanitation, and women’s educational attainment) and a total of 90 independent variables, i.e., the landscape metrics described in Table 2 (the full list of variables is given in Table S1, see Supplementary Materials).

### 2.3. Statistical Models and Feature Selection

We used four different regression models to assess the significance of the associations between the calculated landscape metrics and diarrhoea, while accounting for the effects of access to water, sanitation, and education, as well as spatial autocorrelation. The input data were standardised with a min/max scaler function, so that the effects of the different features could be compared through their coefficients.

We started by running a feature selection algorithm to identify the most important variables to be included in the regression models. Given the high number of independent variables that were derived from the landscape metrics (*n* = 90), a preliminary feature selection was necessary to avoid multicollinearity and to clarify the scope of the analysis. The feature selection algorithm was composed of two “filters”. The first filter was a stepwise selection, which is a process that adds variables from a predefined list to the model, one-by-one, rechecking at each step the importance of all previously included variables [55]. In other words, the stepwise process combines forward and backward feature selection processes, consisting of iterative linear regressions allowing to identify the “best” features based on predefined significance thresholds (maximum *p*-value was set to 0.1) and model performance (residual sum of squares). The second and final filter was based on Spearman’s rank correlations (ρ): we used the Python package *statsmodels* [47] to calculate bivariate correlations between each feature selected with the stepwise method and the dependent variable (i.e., prevalence of diarrhoea at cluster level); only features with a *p*-value smaller than 0.1 were kept. We purposively opted for relative high thresholds of *p*-values because of the exploratory nature of this study.

Once we concluded the feature selection, we ran both weighted and unweighted regression models. First, we built an unweighted ordinary least squares (OLS) model containing the dependent, control, and independent variables, as well as a constant. Then, we built a weighted model with the same features using the cluster weights given by the DHS. In fact, when conducting country-level analyses, the DHS suggests using cluster weights to adjust for eventual biases resulting from their sampling method. Given the infectious nature of diarrhoeal diseases, the analysis also needed to account for spatial dependence [56]. To this end, we used the Python package *pysal* [48] to run two models of spatial regression with the same features, spatial lag and spatial error, as explained in Section 2.4.

### 2.4. Addressing Spatial Dependence

Spatial dependence, or spatial autocorrelation, is the phenomenon by which values of observations are associated with each other through geographic distance (e.g., high values close to other high values) [57]. Accounting for spatial dependence is essential because linear regressions assume a normal, random distribution of error terms and the absence of spatial autocorrelation in the dependent variable. We estimated the probability of spatial autocorrelation in the dependent variable by calculating the global Moran’s *I*, which indicated whether the observed values of prevalence of diarrhoea were clustered, or randomly distributed, in space. As for the error terms in the OLS regression, we detected the probability of spatial dependence through the Lagrange multiplier test for spatial error.

Contrary to an OLS model, spatial regressions can account for the spatial autocorrelation of the dependent variable (spatial lag dependence) and of the error term (spatial error dependence) [58]. The spatial lag model used in this study incorporates the spatial autocorrelation of the dependent variable by introducing the average values of neighbours as an additional variable into the regression specification (Equation (1)):

y = α + ρWy + Xβ + ε(1)

where y is an N × 1 vector of observations on a dependent variable taken at each of N locations, α is the intercept, ρ is a scalar spatial lag parameter, W is an N × N matrix of weights indicating the spatial framework of neighbourhood effects among the dependent variable, X is an N × k matrix of explanatory variables, β is a k × 1 vector of parameters, and ε is an N × 1 vector of error terms. Similarly, the spatial error model used in this study also incorporates spatial autocorrelation by introducing the average values of neighbours as an additional variable into the regression specification, but this time using the values of the error terms (Equation (2)):

y = α + Xβ + u,     u = λWu + ε(2)

where u is the vector of spatially autocorrelated residuals with constant variance and covariance terms, specified by an N × N matrix of weights indicating the spatial framework of neighbourhood effects among the error terms (W) and a spatial error coefficient (λ).

### 2.5. Inclusion Criteria and Stratification of Analysis

The unit of analysis was the buffer area generated from each DHS cluster’s centroid (spatial unit). Out of the 351 spatial units, 10 were excluded as they did not have valid geographic coordinates. Furthermore, because our analysis focused on human settlements, we opted to keep only those spatial units with at least 1 “urban” pixel (300 × 300 m). Hence, we excluded 74 spatial units where no human occupation was detected—including units with settlements not sufficiently large to be detected at the spatial resolution used here. In the end, 267 spatial units (out of 351) were included in our regression analyses. Details about the discarded units are given in Table A1 (Appendix B).

**Figure 4:**
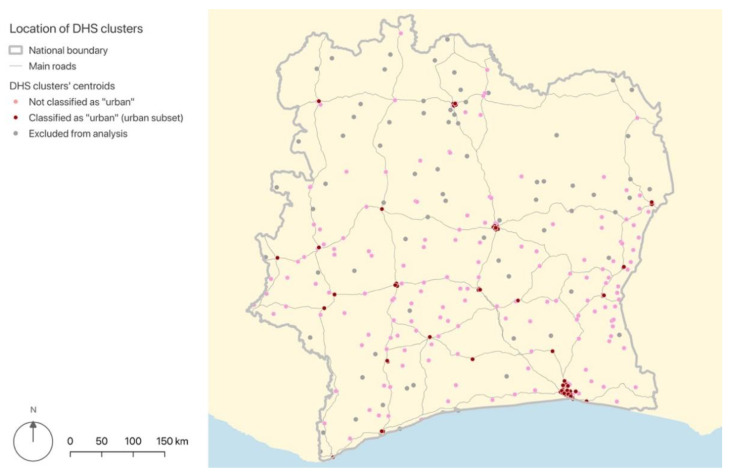
Location of DHS clusters, by subset. Elaborated by the authors with QGIS, from: DHS [28], GADM and OpenStreetMap.

To determine whether the size and proportion of urban areas affected the association between landscape features and diarrhoea, the processes described in Section 2.3 were stratified into two levels. First, we conducted the regression analyses with all the 267 spatial units that met our inclusion criteria. Then, we conducted the same analyses with an “urban” subset, which contained 105 spatial units. The criterion for a spatial unit to be classified as “urban” was to have a proportion of urbanised area (ratio between the surface of “urban” pixels and the spatial unit’s total area) that was above the average of the retained 267 spatial units. Figure 4 shows the location of the 267 spatial units included in the analysis, specified by subset.

## 3. Results

### 3.1. Overall Clustering of Data and Need for Spatial Regressions

The tests for spatial dependence confirmed the need for spatial regression models when processing the data of the full dataset (*n* = 267). The global Moran’s *I* statistics for the spatial distribution of the dependent variable showed a significant, positive spatial autocorrelation of the prevalence of diarrhoea (Moran’s *I* = 0.11, *p* = 0.002). Regarding the distribution of error terms in the OLS regression, a significant spatial dependence was detected by the Lagrange multiplier test (*p* = 0.002). For the urban subset (*n* = 105), however, no spatial dependence was detected. Nevertheless, we also ran spatial regressions with this subset, for the sake of comparison with the full dataset analysis, and overall coherence in the analysis.

### 3.2. Significant Landscape Feature: Dense, Precarious Urban Areas

One feature passed our selection filter and, hence, was retained as an independent variable: the proportion of urban patches (ratio between patch area and buffer area) that were characterised as being very dense (demographic density values situated within the last decile of the series) and with low-to-medium night illumination levels (values above the median but below the last decile of the series, see Figure A1, Appendix A). Following the rationale explained in Section 2.2, these patches correspond to “precarious” urban areas, as the level of density is higher than the illumination level. A total of 44 spatial units (all located around Abidjan and San Pédro, two large cities) had at least one urban pixel with these characteristics. We denominated this type of urban patch as “dense, precarious urban areas”.

Table 3 and Table 4 summarise the results of the different regression models used, following the stratification explained in Section 2.5. The proportion of dense, precarious urban areas was included as single independent variable (as it was the only significant landscape metric), while indicators of access to water, sanitation, and education were included as control variables.

For the full dataset (*n* = 267 spatial units), the weighted OLS regression and the spatial lag model performed better than the other two, based on their R^2^ and Akaike information criterion (AIC) values. Two features consistently showed significant coefficients (*p* < 0.05): (i) proportion of the cluster population with access to basic sanitation facilities; and (ii) proportion of dense, precarious urban areas. In the four models, basic sanitation was negatively associated with diarrhoea, while dense, precarious urban areas showed a positive association and had the strongest beta coefficients. The proportion of women without any education showed significant coefficients in the weighted OLS regression model and, globally, was negatively associated with the prevalence of diarrhoea. There was no significant association between access to basic water facilities and diarrhoea. We observed that neither the directions nor the levels of association between diarrhoea and the tested variables changed significantly between the different models. Also, although spatial dependence was detected, the spatial lag model only improved the coefficient of determination when compared to the OLS model, while it was the weighted model using DHS cluster weights that explained most of the variance of diarrhoea.

For the urban subset (*n* = 105 spatial units), there was considerably less variation in the models’ R^2^ and AIC values. In terms of the explained variance of the dependent variable, the models performed better with the urban subset than the full dataset. In this subset, however, the only feature that consistently showed significant coefficients was the proportion of dense, precarious urban areas—having once again the strongest beta coefficients. Except for sanitation, the control variables showed poor coefficients for the urban subset. As in the full dataset analysis, neither the directions nor the levels of association between diarrhoea and the tested variables changed significantly between the different models. Similarly, the model that explained most of the variance of diarrhoea was the weighted OLS model (with DHS cluster weights).

### 3.3. Stages of Urbanisation and Landscape Patterns

Our stratified regression analyses showed that the proportion of urbanised area within the spatial units affected the coefficients of the selected landscape metric, as well as those of the control variables. Indeed, in the urban subset, the independent variable’s coefficients became more important and significant, while the coefficients of the control variables became much less significant than in the analyses with the full dataset.

**Figure 5:**
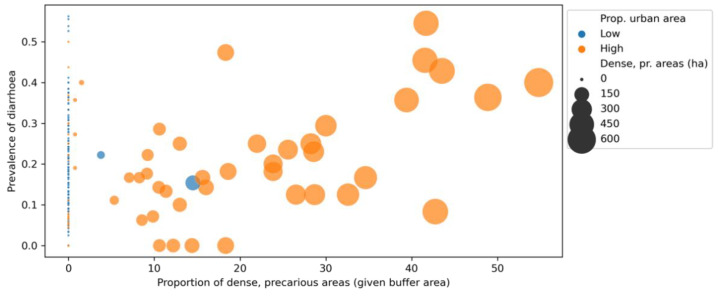
Relation between dense, precarious urban areas (*x*-axis) and the prevalence of diarrhoea (*y*-axis), by level of urbanisation (urban subset shown in orange). Elaborated by the authors.

In addition to the regression coefficients, urbanisation also affected the independent variable’s rank correlation with the outcome of interest (Figure 5). For the full dataset (*n* = 267), the Spearman’s correlation coefficient, which was calculated between dense, precarious urban patches and the prevalence of diarrhoea, was +0.11 (*p* = 0.079), while for the urban subset (*n* = 105; see orange dots in Figure 5) it increased to +0.24 (*p* = 0.013).

The size of urbanised area (total “urban” pixels counted in each buffer area) was positively correlated with the percentage of urban patches that were characterised as dense and precarious (ρ = +0.50, *p* < 0.001). Put differently, as the extent of urbanised areas within the spatial units (buffer areas) increased, the probability of urban areas being classified as “dense and precarious” also increased. Moreover, the cumulated area covered by urban patches was also significantly correlated to the percentage of women without any education (ρ = −0.35, *p* < 0.001), access to basic water facilities (ρ = +0.53, *p* < 0.001), and to basic sanitation (ρ = +0.50, *p* < 0.001).

**Figure 6:**
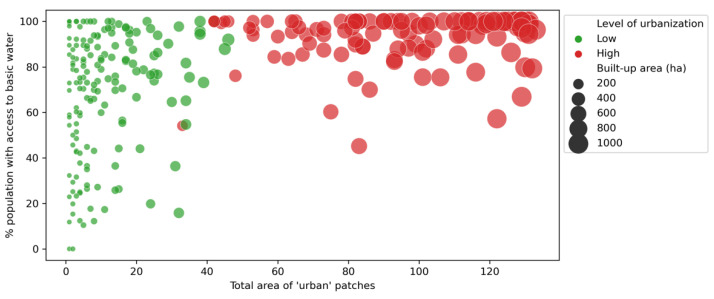
Relationship between the size of the urbanised area (*x*-axis) and access to basic water facilities (*y*-axis), by level of urbanisation (urban subset shown in red). Elaborated by the authors.

**Figure 7:**
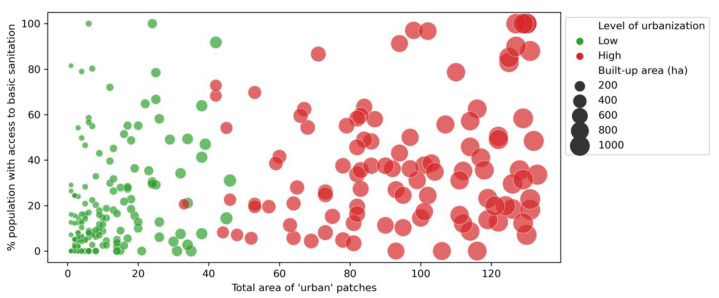
Relationship between the size of the urbanised area (*x*-axis) and access to basic sanitation facilities (*y*-axis), by level of urbanisation (urban subset shown in red). Elaborated by the authors.

**Figure 8:**
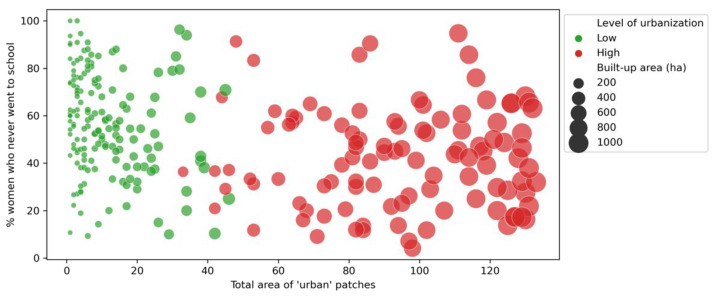
Relationship between the size of the urbanised area (*x*-axis) and the percentage of women without any education (*y*-axis), by level of urbanisation (urban subset shown in red). Elaborated by the authors.

The correlation between urbanisation and access to basic sanitation facilities is less clear than with the other two control variables (Figure 6, Figure 7 and Figure 8). Additionally, the correlations changed according to the stage of urbanisation: we found higher significance levels (*p*-values) for Spearman’s correlations among spatial units having low proportions of urban areas, as compared to the urban subset. We found *p* = 0.002 for water (against 0.318 for the urban subset); *p* ≈ 0.000 for sanitation (against 0.089); *p* = 0.002 for women’s education (against 0.939).

## 4. Discussion

### 4.1. Towards Spatial Predictors of Health Outcomes in Urban Areas

Spatial predictors of socio-economic indicators based on the morphology of the built environment have already been studied [59,60]. At the same time, socioeconomic indicators have been assessed as potential risk factors for diarrhoeal diseases [53]. However, to our knowledge there are no studies that explore direct associations between the urban landscape and diarrhoea. In this sense, our study advanced a selection of landscape metrics as potential predictors of disease. One must note that this research for spatial predictors of diarrhoea was facilitated by the availability of open-source software and open-access data on human settlements and populations, which should be encouraged and valorised both by scientists and planning authorities.

We defined “dense, precarious areas” through demographic and night illumination data. Although hypothetical, this initial assumption proved to be useful in identifying potential vulnerabilities in terms of health. The coefficients of both linear regressions and Spearman’s rank correlation consistently showed a positive, significant association between “dense, precarious areas” and the prevalence of diarrhoea. Among the most urbanised clusters (“urban” subset), the selected landscape metric was a better predictor of the prevalence of diarrhoea than the “usual suspects”, such as access to basic water and sanitation facilities, or women’s educational attainment. In fact, the proportion of dense, precarious urban areas was the only feature to consistently show significant coefficients in all regression models.

These observations call for further investigations on predictors of diarrhoea that are more related to global aspects of the urban habitat, and not strictly focused on household-level indicators or facilities. Indeed, in the case of Côte d’Ivoire, we may argue that poor urban development has led to environmental conditions that limit the potential benefits of basic water and sanitation infrastructures. Without proper urban habitats, the sole presence of these infrastructures may not be sufficient to prevent diarrhoeal diseases. If this is confirmed, it would have policy implications, as decision-makers should aim to improve the overall urban habitat—instead of punctually improving infrastructures, as it may be the case in “slum upgrading” projects. Further research on this aspect could be useful to shed light on recent controversies regarding the health impacts of WASH interventions, which are not always so clear or significant as expected [61].

Although urbanisation in Côte d’Ivoire was correlated to key predictors of diarrhoea, such as the access to basic sanitation and the proportion of dense, precarious areas, it was not directly correlated with diarrhoea. In fact, neither the size nor the form of urban patches showed significant coefficients. Generally, there was no apparent association between city size and the prevalence of diarrhoea, based on aggregated cluster data (Table A2, Appendix C). Nevertheless, the “quality” of urbanisation (indicated here by the extent of “precarious” areas) was a significant feature.

### 4.2. Saturation of Urban Settlements and Health Inequities

Urbanisation has been associated with better aggregate indicators of social and health outcomes [8,62,63]. In Côte d’Ivoire, our results raised some nuances to this assumption. While access to basic water and sanitation, as well as access to education, were significantly associated with urbanisation, the latter alone was not associated in any way with the health outcome of interest—i.e., diarrhoea. On the contrary, depending on the urban landscape characteristics (e.g., the proportion of dense, precarious areas), the risk of diarrhoeal diseases appeared to be significantly higher.

Moreover, our results showed that the size of urban areas was positively associated with the percentage of urban patches that were characterised as dense and precarious. This suggests that infrastructures have failed to keep up with demographic growth, which visibly poses considerable challenges to town planners. The concentration of demographic growth in a few Ivorian cities, and their subsequent saturation, has been discussed previously [23]. As economic limitations hamper investment in infrastructures and access to adequate housing [64], the urban habitat becomes a risk factor for disease. If spatial development is not equitable, then urbanisation leads to health inequities that inflict a high burden on the most deprived populations—notably those living in slums [9,19].

Our analysis also showed that, at early stages of urbanisation, access to basic services significantly increases with urban growth; however, when the extent of urban areas reaches a larger size, this correlation disappears. An explanation could be that it is easier to expand infrastructures in smaller cities than in bigger cities; in the latter, demographic densities would tend to increase without a corresponding increase in infrastructures. In fact, all urban pixels reclassified as “dense, precarious urban areas” were concentrated in two of Côte d’Ivoire’s largest cities, namely Abidjan and San Pédro.

Africa is characterised by the fastest urbanisation rate in the world, and most of the future urbanisation is expected to occur on this continent [21]. Given the important spatial and social transformations that this engenders, specific attention of researchers and urban planners is needed to ensure that the urban environment becomes a catalyst for social development, rather than a health hazard. To this end, early interventions are key to prevent urban areas from becoming saturated and to ensure that basic infrastructures and services keep up with demographic growth. In this sense, focusing on small- and medium-sized cities would be crucial as they approach the “tipping point” (in terms of size of urban area), where it becomes more challenging to expand infrastructures to follow demographic (and spatial) growth.

### 4.3. Study’s Limitations and Need for Further Research

Our study has several limitations that are offered for consideration and, possibly, point towards paths for future research on spatial predictors of diarrhoea. First, the cross-sectional design is susceptible to what is known as the ecological fallacy [65], i.e., limitations to causal inference due to the use of aggregate data. Although precarious spatial development was associated with diarrhoea, there are other features in the complex urban system that may impact the prevalence of diarrhoea. We addressed these limitations by adding control variables (to mitigate effects of confounders) and by reducing the number of independent variables (which reduced multicollinearity in the statistical models).

Second, study design limitations impacted the prediction power of the regression models, which had R^2^ values situated between 0.06 and 0.20. However, we note that low R^2^ values are expected in such ecological studies, given the “noise” of environmental variables and the complexity of the studied ecosystems [66]. In this regard, our model focused on the material aspects of the urban environment, while omitting social dynamics that could be relevant to explain the prevalence of diarrhoea. For instance, mobility can be a key variable to model diarrhoeal diseases, as pathogens may travel long distances with humans [67], but this could not addressed here due to the lack of data. Third, although the stepwise feature selection process is a powerful screening tool to identify contender models, it is prone to statistical issues that merit acknowledgment. For instance, searching through a large number of potential features and keeping only the ones that best fit the sample data may lead to “overfitting” the model [55]. In addition, the multiple testing of a large number of contender features makes such selection methods prone to the “false discovery” of features that appear as significant, but in reality are not relevant to estimate the dependent variable [68]. In this sense, the explanatory variable in this study could be further assessed using datasets with a higher spatiotemporal resolution, based on a larger, random populational sample.

Lastly, the study material was strictly limited to open-access data, and hence, there were limitations in terms of spatial accuracy. While settlement data can be found at a fine spatial resolution, between 100 and 500 m (and potentially up to 0.5 m in the case of very-high resolution satellite imagery), socio-demographic data are only available at low spatial resolutions (in this case, 2 to 5 km). This is certainly due to the ethical considerations regarding privacy. Indeed, researchers must preserve the anonymity of their study participants, and hence, any georeferenced data must be transformed with a geographic blur so that the specific locations of participating households cannot be identified. In our study, this blur made it impossible to determine in which specific type of landscape patch the study subjects lived—which is why we worked with buffer areas and global landscape metrics. Moreover, we note that the socio-demographic data aggregated by clusters came from samples that were not necessarily representative of that same cluster’s population.

Further research is needed to verify to which extent the urban landscape effectively impacts the risk of diarrhoea. Similar study designs could be implemented, but this time using high-resolution socio-demographic data—the latter would certainly need to be primary data collected through household surveys. More generally, further research addressing WASH interventions and diarrhoea from an ecological perspective—i.e., focusing not only on household-level indicators, but also on features observed at community and urban scales—could significantly contribute to fill in the knowledge gaps regarding the health impacts (and efficiency) of different WASH solutions.

## 5. Conclusions

In this exploratory study, we addressed diarrhoeal diseases from a landscape ecology perspective by considering the physical environment as a key explanatory variable. We presented a framework strictly based on secondary, open-access data to assess whether specific patterns of urban landscapes could be associated with diarrhoea. We combined remotely sensed data from different sources to classify urban areas based on demographic density and the intensity of night illumination (used as a proxy for the presence of infrastructures). This allowed us to identify different types of “precarious” urban areas, and to confront them, along with other landscape metrics, with the prevalence of diarrhoea among children under the age of five years.

Based on the results of four different regression models, we found that patches of dense, precarious urban areas were a statistically significant feature, consistently showing a positive association with diarrhoea. This association was stronger in larger urban settlements, where the selected landscape metric was a better predictor of the outcome of interest than the control variables (water, sanitation, and education level). Given these results, we may argue that poor urban development may lead to environmental conditions that hamper the potential benefits of basic water and sanitation infrastructures—thus reducing their ability to prevent diarrhoeal diseases. These observations raise the question of the scope of urban interventions, which should focus on the overall quality of the landscape, and not be limited to punctual infrastructural improvements.

We acknowledge the experimental nature of the framework put forward by this study. There is a need for further research on how the urban environment may be associated with diarrhoea, and how it may impact on potential benefits of water and sanitation infrastructures. Urban planners and public health professionals will benefit from a better understanding of how WASH services interact with their spatial and social contexts, which certainly requires design adaptations.

## Figures and Tables

**Table 1:** List of open-access datasets used to explore relations between landscape and diarrhoea in Côte d’Ivoire.

Data Layer	Source	Description	Available Years	Spatial Resolution	Type
Geolocation of DHS cluster	DHS	Cluster location with a geographic blur of 2 to 5 km	1998/1999 2011/2012	2 to 5 km	Vector (shp ^1^)
Cases of diarrhoea	DHS	Cases of diarrhoea (under-5), geocoded to cluster location	1998/1999 2011/2012	2 to 5 km	Vector (table)
Access to water and sanitation	DHS	Type of facility used by household, geocoded to cluster location	1998/1999 2011/2012	2 to 5 km	Vector (table)
Education attainment	DHS	Education attainment of women (15–49 years), geocoded to cluster location	1998/1999 2011/2012	2 to 5 km	Vector (table)
Climatic conditions	Terra-climate	Accumulated precipitation and mean temperature	1958–2020	1/24th degr. (~4 km)	Raster
Illumination (night lights)	NASA	Intensity of night illumination	2012 & 2016	500 m	Raster
Land use	ESA Land Cover CCI	Discrete categories of land cover	1992–2019	300 m	Raster
Population density	WorldPop	Estimated demographic densities (WorldPop’s model)	2000–2020	100 m	Raster
Roads	OpenStreetMap	Surveyed roads and pathways	2019	5 to 20 m	Vector (shp ^1^)

^1^ shapefile.

**Table 2:** List of variables calculated for each spatial unit (variables aggregated by buffer area).

Variable	Role in Analysis	Aggregation Operation
Prevalence of diarrhoea, under −5	Dependent variable	$\frac{N_{cases^{}}}{N_{pop.\;\;under-5^{}}}$
% access to basic ^1^ water	Control variable	$\frac{N_{pop.\;\;with\;basic\;w.^{}}}{N_{total\;pop.^{}}}$
% access to basic ^1^ sanitation	Control variable	$\frac{N_{pop.\;\;with\;basic\;s.^{}}}{N_{total\;pop.^{}}}$
% women ^2^ with no education	Control variable	$\frac{N_{pop.\;\;women\;no\;ed.^{}}}{N_{pop.\;\;women^{}}}$
Edge of land cover patches ^3^	Independent variables	Total length (m) of edges of given land cover
Shape index of land cover patches ^3^	Independent variables	$\frac{\sum_{i}^{n}P_{area^{i}}\times P_{shp\;index^{i}}}{N_{total\;patches^{}}}$
Proportion of land cover patches ^3^	Independent variables	$\frac{N_{pixels\;of\;given\;land\;cover^{}}}{N_{total\;pixels^{}}}$
% dense urban areas	Independent variable	$\frac{N_{urb.\;\;pix.\;value>median\;urb.\;den.^{}}}{N_{total\;urban\;pixels^{}}}$
% precarious urban areas	Independent variable	$\frac{N_{precarious\;urb.\;\;pixels^{}}}{N_{total\;urban\;pixels^{}}}$
Km of roads per urban area	Independent variable	$\frac{Length\;Rd_{km}}{Urban\;Area_{pixels^{}}}$

^1^ As defined by the WHO and UNICEF Joint Monitoring Programme. ^2^ Aged between 15 and 49 years. ^3^ Applied to all land cover categories.

**Table 3:** Regression results of four models for the full dataset (*n* = 267 spatial units), by model type.

Included Features	Variance Inflation Factor	Unweighted OLS R^2^ = 0.06/AIC = −50.4 JB ^2^: 12.020 (*p* = 0.003) BP ^3^: 4.880 (*p* = 0.300)			Weighted OLS R^2^ = 0.129/AIC = 21.75 JB ^2^: 10.676 (*p* = 0.005) BP ^3^: 2.881 (*p* = 0.578)			Spatial Lag Pseudo R^2^ = 0.09/AIC = −56.3 JB ^2^: 11.407 (*p* = 0.003) BP ^3^: 5.807 (*p* = 0.214)			Spatial Error Pseudo R^2^ = 0.059/AIC = −58.2 JB ^2^: 11.859 (*p* = 0.003) BP ^3^: 4.693 (*p* = 0.320)		
		Coef.	SE	Prob.	Coef.	SE	Prob.	Coef.	SE	Prob.	Coef.	SE	Prob.
Constant	-	0.426	0.070	0.000	0.460	0.066	0.000	0.297	0.076	0.000	0.366	0.073	0.000
% basic water	1.372	0.001	0.063	0.986	0.014	0.062	0.820	0.032	0.061	0.606	0.049	0.064	0.439
**% basic sanitation**	**1.810**	**−0.186**	**0.069**	**0.007**	**−0.257**	**0.069**	**0.000**	**−0.181**	**0.067**	**0.007**	**−0.166**	**0.068**	**0.015**
% women with no ed.	1.674	−0.141	0.075	0.061	−0.221	0.076	0.004	−0.123	0.073	0.092	−0.108	0.074	0.148
**Dense, prec. areas ^1^**	**1.074**	**0.257**	**0.081**	**0.002**	**0.291**	**0.062**	**0.000**	**0.227**	**0.081**	**0.005**	**0.275**	**0.094**	**0.004**

^1^ Selected landscape metric: proportion of dense, precarious urban areas (total patch area/buffer area). ^2^ Jarque-Bera test for normality of errors. ^3^ Breusch-Pagan test for heteroskedasticity. Bold characters indicate that the association is significant (*p* < 0.05).

**Table 4:** Regression results of four models for the urban subset (*n* = 105 spatial units), by model type.

Included Features	Variance Inflation Factor	Unweighted OLS R^2^ = 0.141/AIC = −14.04 JB ^2^: 4.316 (*p* = 0.116) BP ^3^: 4.444 (*p* = 0.349)			Weighted OLS R^2^ = 0.196/AIC = 23.56 JB ^2^: 6.395 (*p* = 0.041) BP ^3^: 7.101 (*p* = 0.131)			Spatial Lag Pseudo R^2^ = 0.141/AIC = −12.05 JB ^2^: 4.364 (*p* = 0.113) BP ^3^: 4.391 (*p* = 0.356)			Spatial Error Pseudo R^2^ = 0.141/AIC = −14.09 JB ^2^: 4.285 (*p* = 0.117) BP ^3^: 4.424 (*p* = 0.352)		
		Coef.	SE	Prob.	Coef.	SE	Prob.	Coef.	SE	Prob.	Coef.	SE	Prob.
Constant	-	0.282	0.137	0.042	0.325	0.157	0.040	0.290	0.143	0.043	0.275	0.134	0.041
% basic water	1.172	0.082	0.118	0.488	0.112	0.136	0.412	0.081	0.115	0.483	0.089	0.115	0.439
% basic sanitation	**1.769**	−0.168	0.107	0.119	**−0.252**	**0.107**	**0.021**	−0.167	0.105	0.111	−0.168	0.105	0.110
% women with no ed.	1.810	−0.013	0.127	0.917	−0.088	0.136	0.520	−0.012	0.124	0.925	−0.012	0.124	0.922
**Dense, prec. areas ^1^**	**1.029**	**0.315**	**0.090**	**0.001**	**0.316**	**0.079**	**0.000**	**0.318**	**0.093**	**0.001**	**0.318**	**0.090**	**0.000**

^1^ Selected landscape metric: proportion of dense, precarious urban areas (total patch area/buffer area). ^2^ Jarque-Bera test for normality of errors. ^3^ Breusch-Pagan test for heteroskedasticity. Bold characters indicate that the association is significant (*p* < 0.05).

## Data Availability

Data available in a publicly accessible repository are listed below. Data presented in this study (processed by the authors, i.e., buffers with aggregated household and landscape data) will openly available in Zenodo. Until the publication, they will be provisionally available on Git Hub, with the computer code (see “Computer Code and Software”). DHS data: restrictions apply to the availability of these data. Data was obtained from the Demographic and Health Surveys Program and are available at https://dhsprogram.com/data/dataset/Cote-d-Ivoire_Standard-DHS_2012.cfm?flag=0 (accessed on 11 May 2022) with the permission of the Demographic and Health Surveys Program. ESA Land Cover CCI data: restrictions apply to the availability of these data. Data was obtained from ESA Land Cover CCI and are available at https://maps.elie.ucl.ac.be/CCI/viewer/download.php (accessed on 11 May 2022) with the permission of ESA Land Cover CCI. NASA: publicly available datasets were analysed in this study. This data can be found here: https://earthobservatory.nasa.gov/features/NightLights/page3.php (accessed on 11 May 2022). OpenStreetMap: publicly available datasets were analysed in this study. This data can be found here: https://download.geofabrik.de/africa.html (accessed on 11 May 2022). Terraclimate: publicly available datasets were analysed in this study. This data can be found here: https://www.climatologylab.org/terraclimate.html (accessed on 11 May 2022). WorldPop: publicly available datasets were analysed in this study. This data can be found here: https://www.worldpop.org/project/categories?id=3 (accessed on 11 May 2022).

## Supplementary Materials

The following supporting information can be downloaded at: https://www.mdpi.com/article/10.3390/ijerph19137677/s1, Table S1: Spearman correlation coefficients between all environmental variables (*n* = 90) and the prevalence of diarrhoea, with the respective *p*-value (for each variable, the table also indicates whether it was selected by the stepwise regression).

## Computer Code and Software

A repository with the code materials used in this study is available at https://github.com/ceat-epfl/landscape-diarrhoea-civ (accessed on 28 May 2022).

## Appendix A

Figure A1 illustrates the rationale behind the reclassification of urban pixels (300 × 300 m), based on the levels of demographic density and night illumination. Three thresholds were established for these two statistical series, corresponding to: (i) first decile; (ii) median; (iii) last decile. These thresholds allowed to classify each pixel into 4 classes of demographic density and night illumination (1 to 4, with “4” corresponding to the highest values, i.e., within the last decile of the series). Urban pixels with a density class higher than its illumination class were considered “precarious”.

## Appendix B

Table A1 shows the general characteristics of the excluded and retained spatial units, based on the variables provided by the DHS surveys. The reader must note that it was not in the scope of this study to analyse these differences. In fact, our analysis focused on human settlements, which requires the presence of urban land cover “patches”—which is why we only included spatial units containing at least 1 pixel (300 × 300 m) classified as “urban”.

Globally, the 84 units that did not meet the inclusion criteria (explained in Section 2.5) showed similar prevalence rates of diarrhoea, as well as access to basic water facilities, to the 267 units retained for the analysis. As for the levels of access to basic sanitation and women’s education, the values were significantly different.

**Table A1:** General characteristics of the excluded and retained spatial units (obtained from DHS data).

Statistic	Prevalence of Diarrhoea (%)		Access to Basic ^1^ Water (%)		Access to Basic ^1^ Sanitation (%)		Women who Never Went to School (%)	
	Excluded obs. (*n* = 84)	Retained obs. (*n* = 267)	Excluded obs. (*n* = 84)	Retained obs. (*n* = 267)	Excluded obs. (*n* = 84)	Retained obs. (*n* = 267)	Excluded obs. (*n* = 84)	Retained obs. (*n* = 267)
Median value	16.7	16.7	67.1	87.4	3.7	18.5	83.0	50.0
Mean value	17.8	18.2	61.0	78.1	6.2	27.0	80.1	51.0
Standard deviation	10.9	12.6	27.7	24.9	7.9	26.1	15.4	22.2
Minimum value	0.0	0.0	0.0	0.0	0.0	0.0	34.8	4.2
Maximum value	42.9	56.3	100.0	100.0	33.3	100.0	100.0	100.0

^1^ Definitions of “basic” and “safe” WASH facilities based on the WHO & UNICEF Joint Monitoring Programme.

## Appendix C

Table A2 shows aggregated DHS data for a selection of cities across Côte d’Ivoire. These cities were selected based on their population sizes and, also, based on their role as “connectors” within the urban system of Côte d’Ivoire [23]. The data was aggregated spatially: the mean cluster-level prevalence of diarrhoea was calculated by selecting the specific clusters that were located in each city. There was no apparent association between city size (suggested here by the number of clusters comprised in each city) and the mean prevalence of diarrhoea; although Abidjan showed the highest maximum value (54.5%), small- and medium-sized towns such as Daloa and Douékoué showed much higher, mean prevalence rates of diarrhoea (26.0 and 43.8%, respectively).

**Table A2:** Observed prevalence of diarrhoea among children under five in a selection of Ivoirian cities.

Location	Size and Category ^1^	N° Clusters	Mean Prevalence of Diarrhoea ^2^	Standard Deviation of Sample	Range (Min. and Max. Values)
Abidjan	Large (“global connector”)	48	21.4	13.8	0.0/54.5
Yamoussoukro	Medium (“global connector”)	5	14.5	11.0	0.0/29.4
San Pédro	Medium (“global connector”)	4	14.5	13.2	0.0/28.6
Bouaké	Medium-large (“regional connector”)	16	12.1	9.4	0.0/33.3
Korhogo	Medium (“regional connector”)	6	12.5	11.5	0.0/29.4
Daloa	Medium (“regional connector”)	4	26.0	7.5	20.0/36.8
Katiola	Small (“local connector”)	2	5.9	8.3	0.0/11.8
Douékoué	Small (“local connector”)	1	43.8	-	-
Divo	Small (“local connector”)	1	15.4	-	-

^1^ “Connector” categories based on the work by Fall and Coulibaly [23]. ^2^ Mean values resulting from aggregated DHS data.

## Appendix D

Table A3 shows the results of the weighted OLS regression done with a pre-selection of control variables. The regression used the cluster weights provided by the DHS and was run with the 267 spatial units included in the study. The pre-selection of control variables was done based on the literature, including features that have been associated with diarrhoeal diseases (as explained in Section 2.2). Considering the extremely poor performance of variables related to climatic conditions and hygiene facilities, we opted to exclude them from the analysis. Only the variables related to water, sanitation and women’s education were retained as control variables.

**Table A3:** Results of the weighted OLS regression models for the pre-selected control variables (full dataset, *n* = 267 spatial units).

Pre-Selected Control Variables	Variance Inflation Factor	Weighted OLS (DHS Cluster Weights) R^2^ = 0.059/AIC = 46.45 Jarque-Bera Test for Normality of Errors: 31.090 (*p* < 0.001) Breusch-Pagan Test for Heteroskedasticity: 4.152 (*p* = 0.656)		
		Coef.	SE	Prob.
Constant	-	0.448	0.079	0.000
% of the population with access to basic water facilities ^1^	1.468	0.074	0.067	0.270
% of the population with access to basic sanitation facilities ^1^	1.970	−0.254	0.076	0.001
% of the population with access to safe hygiene facilities ^1^	1.363	−0.034	0.054	0.535
% of the female population who never went to school	1.744	−0.261	0.079	0.001
Mean accumulated precipitation (monthly values) in 2012	1.260	0.049	0.063	0.436
Mean maximal temperature (monthly values) in 2012	1.165	0.0008	0.091	0.993

^1^ Definitions of “basic” and “safe” WASH facilities based on the WHO & UNICEF Joint Monitoring Programme.
